# Analysis of the Impact of Ethanol Extract of *Calendula officinalis* L. on Human Fibroblast Cell Cultures Using the PANsys 3000 Device for Breeding and Visualization of Cells

**DOI:** 10.3390/life13101949

**Published:** 2023-09-22

**Authors:** Agnieszka Nowak-Terpiłowska, Izabela Nowak, Agnieszka Feliczak-Guzik, Marzena Wyganowska

**Affiliations:** 1 Department of Biochemistry and Biotechnology, Poznan University of Life Sciences, Dojazd 11 St., 60-632 Poznan, Poland; 2 Department of Applied Chemistry, Adam Mickiewicz University, Uniwersytetu Poznańskiego 8 St., 61-614 Poznan, Poland; nowakiza@amu.edu.pl (I.N.); agaguzik@amu.edu.pl (A.F.-G.); 3 Department of Dental Surgery, Periodontology and Oral Mucosa Diseases, Poznan University of Medical Sciences, Bukowska 70 St., 60-812 Poznan, Poland; marzena.wyganowska@periona.pl

**Keywords:** *Calendula officinalis* L., fibroblasts, morphology, proliferation

## Abstract

*Calendula officinalis* L. promotes wound healing and might be effective in gingival fibroblast stimulation. The influence of different concentrations of *Calendula officinalis* L. ethanol extract on human gingival fibroblast was visualized using PANsys 3000—a fully automated cell culture device used for in vitro culture to study cells under conditions similar to in vivo. The human fibroblast cells were isolated from gingival tissue. The 100% brew of *Calendula officinalis* L., as well as 7% and 20% *Calendula officinalis* L. ethanol extract, were added to the cultured cells and observed for 72 h. The qualitative and quantitative composition of the volatile compounds of marigold *Calendula officinalis* L. flowers are presented in this study. The essential oil compounds of the decoction were isolated with solid-phase microextraction (SPME) and analyzed with gas chromatography coupled with tandem mass spectrometry (GC-MS/MS). The presence of terpenoids, flavonoids, and other compounds was demonstrated. The composition was correlated with the fragrance properties. Observation of gingival fibroblast showed that there were no changes in cell morphology and proliferation after 100% *Calendula officinalis* L. brew stimulation. The growth and cell division were not inhibited. Likewise, the addition of 7% or 20% ethanol in water extract of *Calendula officinalis* L. stimulation did not inhibit the fibroblast proliferation. Overall, ethanol extracts of *Calendula officinalis* L. decrease the alcohol cytotoxic influence on gingival fibroblasts.

## 1. Introduction

Traditional medicinal herbs with few side effects have generated a lot of interest in the field of disease treatment or prevention in recent years. Research shows that herbal extracts and compounds have therapeutic effects through the activity of autophagy-enhancing pathways, antioxidant properties, or anti-inflammatory properties [1,2]. *Calendula officinalis* L. (CO, also known as English Marigold, Pot Marigold, Holigold, Mary Bud, Marybud, or Mary Gowles) is a one-year plant and is considered an important plant in traditional medicine. CO is widely used in various therapeutic applications, e.g., pharmacy, cosmetology, and food industries (e.g., a yellow pigment extracted from *C. officinalis* L. flowers is used as a food coloring additive) [3,4]. CO, being a source of secondary metabolites, has anti-inflammatory, antidiabetic, antioxidant, anticancer, antibacterial, antiulcer, antifungal, antiviral, antithrombotic, neuroprotective, antiprotozoal and skin-protecting properties [5]. The genus *Calendula* includes about 25 species, the most common being *C. officinalis*, *C. arvensis*, *C. tripterocarpa*, *C. stellata,* and *C. suffruticose* [6]. CO occurs near warm and humid atmospheric conditions, e.g., in Central European and Mediterranean regions [7,8]. It is also found in Middle Eastern countries, especially Cyprus, Turkey, and Iran. CO has been used in medicine since the 12th century and is the most commonly studied species of marigold [9]. For a long period, this traditional herb has been used to treat minor burns, wounds, and skin problems. Currently used CO medicines include pot marigold tincture and carophyllenic ointment, which both contain carotenoids derived from the flowers. According to the European Medicines Agency, CO oil is classified as a herbal medical product [10,11].

The leaves and stems of the plant contain carotenoids, mostly lutein, zeaxanthin, and beta-carotene. The flowers of *C. officinalis* L. contain flavonol glycosides, triterpene oligoglycosides, oleanane-type triterpeneglycosides, saponins, and a sesquiterpene glucoside. From the phytochemicals identified in the plant, many possess anti-inflammatory activities. *Calendula officinalis* L. is rich in quercetin, carotenoids, lutein, lycopene, rutin, ubiquinone, and xanthophylls. An in vitro study has documented that *Calendula* has anti-inflammatory effects, which are probably related to triterpenoid fatty acid esters, like arnidol, faradiol, and calenduladiol. The most abundant esters of these are lauryl, myristoyl, and palmitoyl esters [12]. The therapeutic effectiveness of a *C. officinalis* L. extract is well-known [13]. In traditional medicine, the water extract of *C. officinalis* L. is usually used; however, as a commercial product, an alcohol extract or essential oil is applied. Water extract of *C. officinalis L.*, in comparison with its hydroethanol extract, significantly stimulates the proliferation and migration of dermal fibroblasts when applied topically on the wound. The effect is dose-dependent. In a 24 h observation, the migration slightly decreased, but the proliferation was still clearly visible. It is possible that only water extract that contains an active component of the plant influences the cells’ reactions. The quercetin 3-*O*-glucoside and rutin are pointed out as potentially active components in *C. officinalis* L. [14]. Alcohol reduced cell viability and increased reactive oxygen species (ROS) significantly in oral fibroblasts [15]. Decreasing cell viability in response to increasing alcohol concentration was dose-dependent. Fibroblasts, cells of mesodermal origin, are the most abundant gingival connective tissue cells. These cells and the extracellular matrix components they produce (collagen, reticulin, elastic fibers, glycoproteins, glycosaminoglycans, cytokines, growth factors, and the enzymes collagenase and stromelysin) play a key role in maintaining connective tissue cohesiveness, healing, and pathological processes [16,17]. These cells express a number of metalloproteinases (MMPs) called matrixins. Matrixins not only play a role in periodontal destruction but also contribute to the development of caries, inflammation of the pulp and periapical tissues, tumors, or lichen planus [18,19]. To date, 28 MMPs have been detected in vertebrate organisms, including 23 in humans. Due to differences in their molecular structure and substrate specificity, MMPs have been divided into six groups: collagenases, which degrade collagens, types I, II, III, VI, and X (MMP-1, -8, -13, -18); gelatinases, which degrade gelatin and type IV collagen (MMP-2, -9); stromelysins, which degrade fibronectin and collagen (MMP-3, -10, -11); matrilysins, which degrade type IV collagen, fibronectin, and fibrinogen (MMP-7, -12, -26); membrane-type metalloproteinases (MT-MMPs), which activate other metalloproteinases (MMP-14, -15, -16, -17, -24, -25); and those not assigned to the abovementioned groups (MMP-12, -19, -20, -21, -22, -23, -27, -28) [20].

An important local mechanism by which alcohol can interfere with fibroblasts in the wound-healing process in maxillofacial injuries is the inhibition of fibroblast proliferation and extracellular matrix (ECM) synthesis at the wound site [21]. Interestingly, a hydroalcoholic extract of *Calendula officinalis* L. was evaluated for acute oral toxicity in rats and mice; subacute effects on hematological, biochemical, and morphological parameters were observed and did not cause death in animals after oral doses of up to 5.0 g/kg. Oral treatment of hydroalcoholic extract of *Calendula* in lower concentrations of 0.025, 0.25, 0.5, and 1.0 g/kg resulted in no hematological changes compared to the control group. In biochemical parameters, an increase in blood urea nitrogen and alanine transaminase was observed. Morphological examination of the brain, kidneys, and heart showed no changes. Hydroalcoholic extract of *Calendula* was nontoxic in rats, although there was evidence of renal and hepatic overload [22]. CO micro- and nano-preparations have excellent potential for treating several ailments, and their future applications will have great impacts. This study aimed to observe the influence of *Calendula officinalis* L. ethanol extract concentrations on human gingival fibroblasts by using PANsys 3000.

## 2. Materials and Methods

Marigold (*Calendula officinalis* L.) was obtained as a gift after the species were validated by the Plant Systems Department of the Botanical Garden of Adam Mickiewicz University in Poznan.

For the experiment, PANsys 3000 (Systech GmbH, Augsburg, Germany) was used. PANsys 3000 is a fully automated cell culture device used for in vitro culture and for studying a variety of cell lines under conditions similar to in vivo. The system offers an automated evaluation of the cells (growth, metabolism, and morphology) and allows the culturing of various cells, while using various compositions of the media at the same time, with any culture conditions and the selected microscopic observation. In the cell culture chambers, the cells are automatically supplied with the corresponding media according to the culture parameters. PANsys 3000 records images of every selectable point of the cell culture according to specified time intervals. All data associated with the experiments can be recorded and played back later.

### 2.1. Analysis of the Decoction/Macerate from Calendula officinalis *L.*

A total of 0.5 g of dried marigold (*Calendula officinalis* L.) was immersed in 25 mL of ethyl alcohol and treated with ultrasound at 30 °C for 1 h. The macerate obtained was filtered and analyzed with GC-MS by direct injection. Additionally, 0.5 g of dried marigold was brewed in 25 mL of water in the same manner as for mouthwashes (decoction, see Section 2.2).

The quantitative and qualitative identification was carried out with GC-(EI)MS/MS (Varian GC4000). The mass spectra were measured over the *m*/*z* range of 10–1000. The identification of components was made by matching their mass spectra with those of reference compounds in the data system of the Wiley Library and NIST Mass Spectral Search Program (2005 Version Database). The volatile flavor components were also matched by co-injection with authentic compounds.

The GC analysis was performed by using an MS-5 capillary column (30 m × 0.25 mm i.d. × 0.25 μm film), and helium was used as a carrier gas (1 mL min^−1^). The initial temperature was programmed from room temperature (RT) to 100 °C (at 2 °C min^−1^), to 170 °C (at 5 °C min^−1^), to 220 °C (at 15 °C min^−1^), and maintained at 220 °C for 5 min.

Four types of SPME fibers coated with different stationary phases (PDMS, PA, PDMS–DVB, and CW–DVB) were used to examine their extraction efficiencies. The solid-phase microextraction fiber coated with polydimethylsiloxane/divinylbenzene (PDMS/DVB) was selected based on a preliminary screening (the highest qualitative, i.e., number of volatiles extracted and quantitative data, i.e., peak areas obtained—data not shown) for the additional analysis of ethanol extract (macerate) and decoction. The fiber was exposed for 2 min at 25 °C for volatile adsorption and then inserted into the injection port of the GC system for thermal desorption and reconditioning (2 min at 280 °C).

### 2.2. Mouthwashes

Herbal decoction of *Calendula officinalis* L. was made in a traditional way: 2 g of *Calendula officinalis* L. herb flowers was brewed in distilled water at the temperature of 90 °C and then boiled under cover for 10 min. The solutions were left in sterile, tightly closed containers until they cooled down. Before the addition to the cell cultures, extracts were filtered on 0.2 μm sterile filters. After 24 h of culturing, the following was produced: for 7 wt.% alcohol concentration in *Calendula officinalis* L. extract, 232.5 μL of herbal decoction was added to 17.5 μL of ethanol PA 96° GL; for 20 wt.% alcohol concentration in *Calendula officinalis* L. extract, 200 μL of herbal decoction was added to 50 μL of ethanol PA 96° GL; and for 100% *Calendula officinalis* L. extract concentration, only herbal decoction was added. Next, a 50% DMSO (dimethyl sulfoxide) solution in DMEM (Dulbecco’s Modified Eagle’s Medium) was added to the cells as a toxic compound, serving as a negative control. DMEM supplemented with 10 wt.% FBS (Fetal Bovine Serum) and 1 wt.% antibiotic-antimycotic solution (10,000 units penicillin, 10 mg streptomycin, and 25 μg amphotericin B per ml) was added to the cells to serve as a positive control.

### 2.3. Cell Culture

This study was conducted with the approval of the bioethics committee (consent no. 150/17 from 2 March 2017 by decision of the Bioethics Commission at the Medical University of Karol Marcinkowski in Poznan) and all subjects gave their informed consent for inclusion before they participated in the study. In order to perform the experiment, a primary cell culture of human gingival fibroblast cells isolated from tissue obtained during a standard surgical procedure was introduced. Tissues were obtained from 5 patients and cultured in tissue flasks with a surface of 25 cm^2^ DMEM supplemented with 10 wt.% FBS and 1 wt.% antibiotic–antimycotic solution at 37 °C and 5% CO_2_. High-confluence cultures were rinsed with Hanks solution and trypsinized (0.25% trypsin and 0.02% EDTA). Cells were gathered by centrifugation (1000 rpm, 10 min). A total of 500 μL of the cell suspension was combined with 500 μL culture medium supplemented with 10% FBS and 1% antibiotic–antimycotic solution and placed in a PANsys 3000 growth chamber device.

### 2.4. Influence of Rinses on Fibroblast Proliferation

Before *Calendula officinalis* L. herbal preparations were added, the cells were removed from TC flasks and transferred to separate cell culture chambers (multi-chamber system) with individual equipment for each chamber and supervision of all relevant cell culture parameters. Next, 250 μL of cell suspension (assessed using Scepter™ 2.0 Cell Counter) was mixed with a medium solution to achieve a concentration of 5 × 10^5^ cells/mL. Cultivation in chambers was conducted 24 h before rinsing stimulations. Consequently, 1.25 mL of cell suspension in the culture medium was supplemented with 250 µL of each rinse. To evaluate the effects of herbal preparations on fibroblasts, the cells were grown for 48 h after addition. Continuous video-microscopic monitoring of the cells was conducted. Morphological changes and growth behaviors were recorded (Videos S1–S5).

## 3. Results

A total of 21 different terpenes were identified in the *Calendula officinalis* L. as volatile compounds (Table 1). Sesquiterpenes were the major chemical class in ethanolic extract and decoction (water extract). The most abundant was α-cadinene (18.1%), followed by δ-cadinene (14.9%) and γ-cadinene (8.9%).

**Figure 1:**
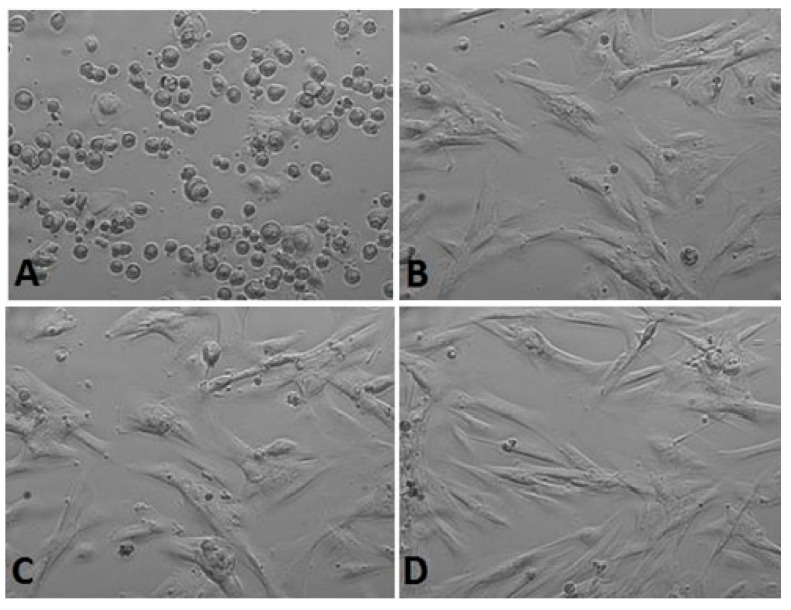
Gingival fibroblasts before 7% alcohol *Calendula officinalis* L. extract addition: (**A**) cells 1 h after the start of examination, (**B**) cells directly before 7% alcohol *Calendula officinalis* L. extract addition and after *Calendula officinalis* L. extract addition, (**C**) cells directly after 7% alcohol *Calendula officinalis* L. extract addition, and (**D**) cells at the end of examination (48 h after extract addiction). Inverted fluorescence microscope, magnification 40×.

**Figure 2:**
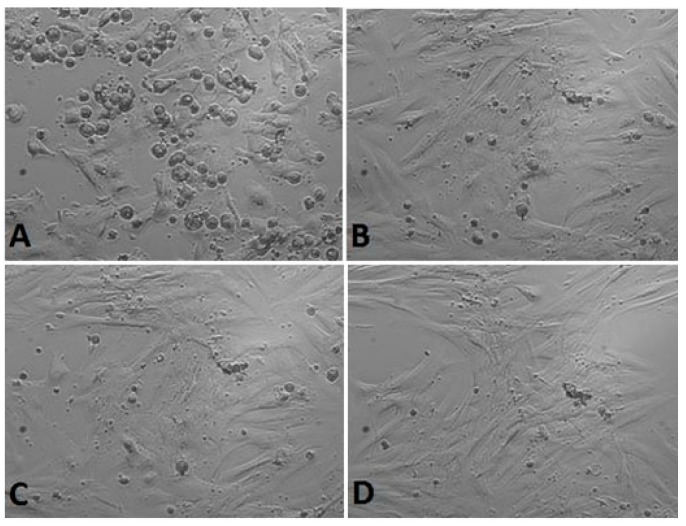
Gingival fibroblasts before 20% alcohol *Calendula officinalis* L. extract addition: (**A**) cells 1 h after the start of examination, (**B**) cells directly before 20% alcohol *Calendula officinalis* L. extract addition and after *Calendula officinalis* L. extract addition, (**C**) cells directly after 20% alcohol *Calendula officinalis* L. extract addition, and (**D**) cells at the end of examination (48 h after extract addiction). Inverted fluorescence microscope, magnification 40×.

**Figure 3:**
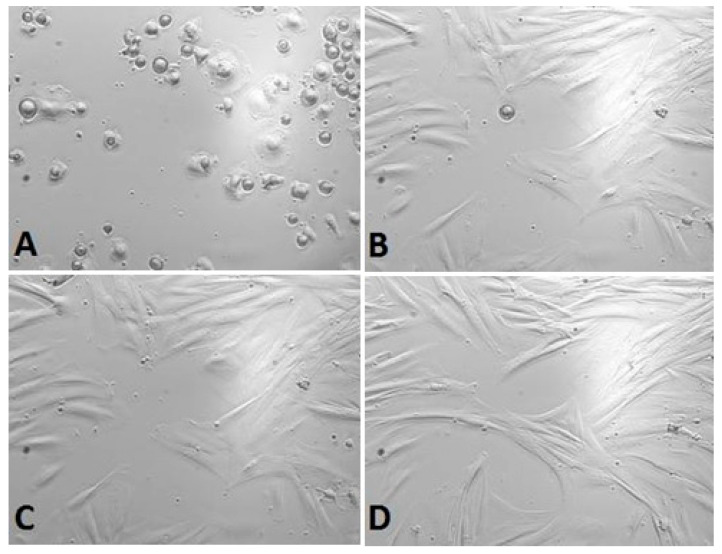
Gingival fibroblasts before 100% *Calendula officinalis* L. extract addition: (**A**) cells 1 h after the start of examination, (**B**) cells directly before 100% *Calendula officinalis* L. extract addition and after *Calendula officinalis* L. extract addition, (**C**) cells directly after 100% *Calendula officinalis* L. extract addition, and (**D**) cells at the end of examination (48 h after extract addiction). Inverted fluorescence microscope, magnification 40×.

**Figure 4:**
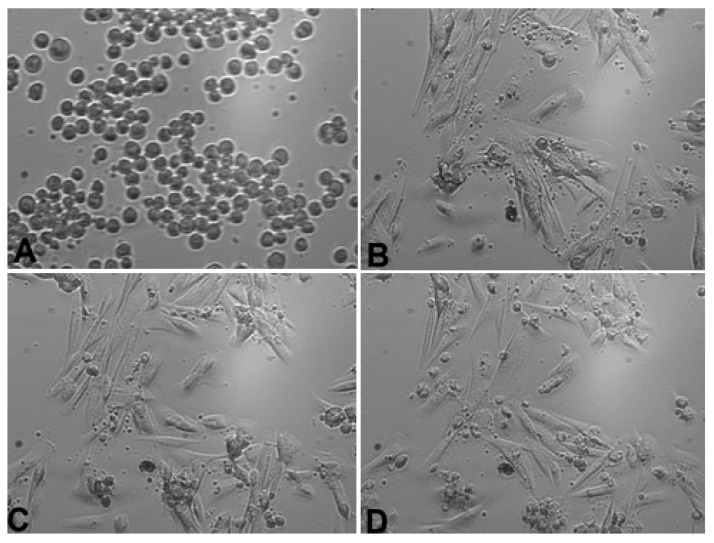
Positive control—gingival fibroblasts without extract addition: (**A**) cells 1 h after the start of examination, (**B**) cells 24 h after the start of examination, (**C**) cells 48 h after the start of examination, and (**D**) cells 72 h after the start of examination. Inverted fluorescence microscope, magnification 40×.

**Figure 5:**
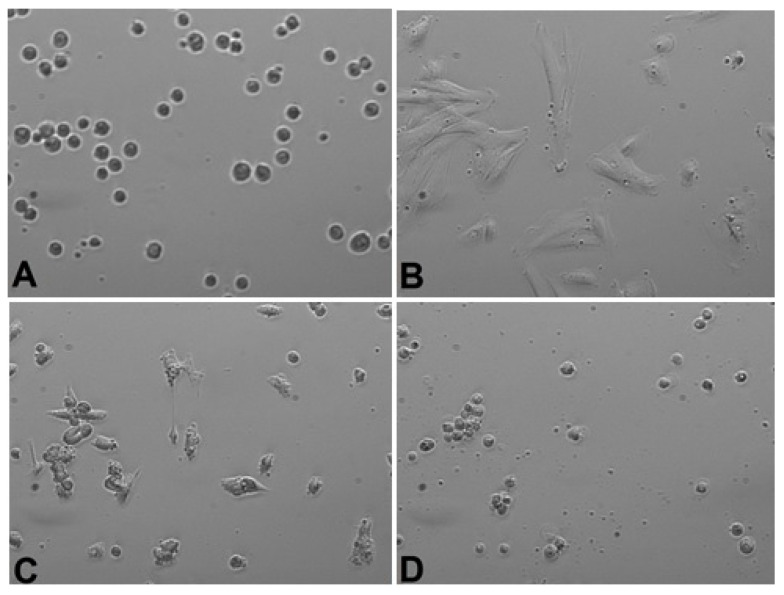
Negative control—gingival fibroblasts with 50% DMSO addition: (**A**) cells 1 h after the start of examination, (**B**) cells 24 h after the start of examination, (**C**) cells 1 h after 50% DMSO addition and 48 h after the start of examination, and (**D**) cells 24 h after 50% DMSO addition and 72 h after starting the examination. Inverted fluorescence microscope, magnification 40×.

*Calendula* decoction did not cause a noticeable decrease in the number of cells in chambers for any examined extracts (Figure 1, Figure 2 and Figure 3). In comparison with the positive control group (gingival fibroblasts without extract addition, Figure 4), both 7% and 20% ethanol in *Calendula officinalis* L. extract and 100% *Calendula officinalis* L. extract without ethanol addition showed no significant differences after 24 and 48 h of culture (Supplementary Materials Videos S1–S3). The morphology of the cells was normal. Inhibition of cell growth and proliferation was observed with the negative control (Figure 5 and Video S5).

*7% ethanol concentration*

*20% ethanol concentration*

*100% Calendula officinalis L. extract*

*Positive control—no extract addition*

*Negative control—50% DMSO addition*

## 4. Discussion

Wound healing is a dynamic and complex process dependent upon the matrix constituents within the wound and cytokines/growth factors produced by inflammatory cells, keratinocytes, and fibroblasts [23]. The mode of action of *C. officinalis L.* tincture on wound healing is poorly understood. *Calendula officinalis* L. is usually used as a water or alcohol extract.

In our previous study, after 24 and 48 h of culturing fibroblasts in a medium with water extract of *Calendula officinalis* L., a gradual increase in the number of cells on the plates was observed. The increase was slightly higher than in the control group: 146.6 ± 35.7% vs. 112.4 ± 1.3% (*p* = 0.14) for the 24 h culture, and 183.2 ± 60.5% vs. 137.4 ± 5.8% (*p* = 0.13) for the 48 h culture, respectively; the difference was not significant. No apoptotic structural changes were observed after 48 h of culture. There were no morphological changes observed. Moreover, when the *Calendula officinalis* L. brew was applied, favorable morphological changes in the cell structure were visible. They were manifested by numerous and elongated projections and an increased number of cells [24]. The same results were also observed with higher dilution (results not shown). Other studies confirmed that extracts of *Calendula officinalis* L. stimulated the proliferation and migration of fibroblasts at low concentrations [25,26].

The other observations connected with the influence of alcohol on fibroblasts led us to the conclusion that both of the contaminations of 7.2% and 22% ethyl alcohol negatively affect the morphology and cell proliferation. An addition of a 7.2% ethanol concentration enabled cells to regain their ability to divide and recover normal morphology after 10 h, whereas changes were irreversible when caused by 22% ethanol [27].

In the current study, ethanol was added to the water extract of *Calendula officinalis* L. to achieve a different concentration. Similar to in vivo observation of gingival fibroblast provided by PANsys 3000, results showed that there were no changes in cell morphology and proliferation after 100% *Calendula officinalis* L. brew stimulation. The growth and cell division were not inhibited. This result confirmed our previous observation. Likewise, after 7% and 20% ethanol concentration was added in water extract of *Calendula officinalis* L. stimulation, fibroblast proliferation was not inhibited. The fibroblast clusters were visible in all pictures captured by the microscope. Hormozi et al. [10] indicated that the 50% alcohol concentration *Calendula officinalis* L. extract stimulated embryonic mouse fibroblast to proliferate via the expression of growth factors (TGFβ1 and bFGF). The authors also compared the impact of different concentrations of *Calendula officinalis* L. added to the 50% alcohol on fibroblast proliferation. The best results were achieved when the 5 μg/mL and 10 μg/mL were used.

Matysik et al. [28], in a study on cell proliferation and cellular metabolism stimulation via an increase in mitochondrial dehydrogenase activity, showed the addition of *C. officinalis* L. extract to heptane, ethyl acetate, and methanol. Only ethyl acetate extracts were in concentrations above 25 μg/mL; however, concentrations exceeding 75 μg/mL were toxic to cells. Moreover, cytotoxicity experiments demonstrated that *C. officinalis* L. hydroalcoholic extract was not cytotoxic to L929 and HepG2 cells at concentrations less than or equal to 15 mg/mL. However, in concentrations greater than or equal to 30 mg/mL, toxic effects were observed. In addition, this extract affects the activity/secretion of matrix metalloproteinases 2 and 9 (MMP-2 and -9) [29].

Amongst three different extracts from *Calendula officinalis* L. flowers (n-hexanic, ethanolic, and aqueous), only n-hexanic and the ethanolic extracts influence the inflammatory phase by activating the transcription factor NF-κB and by increasing the amount of the chemokine IL-8. The ethanolic extract inhibited the activity of collagenase in vitro and enhanced the amount of collagen in the supernatant of human dermal fibroblasts [17].

Quercetin, one of the active components in *Calendula officinalis* L., is suggested to be responsible for inhibiting recombinant human matrix metalloproteinase (MMP) activity. Moreover, it decreases the expression of tumor necrosis factor-α, interleukin-1β (IL), IL-6, and IL-8 in phorbol 12-myristate 13-acetate, and calcium ionophore-stimulated human mast cells, as well as lowers the cytotoxicity of alcohol. Saini et al. indicated, however, that *Calendula officinalis* L. inhibited HGF-mediated collagen degradation and MMP-2 activity more than the same correlated concentration of pure quercetin [30].

In HPTLC with densitometry, except quercetin and isoquercitrin, the following: oleanolic acid, beta-amyrin, beta-amyrin acetate, rutin, narcissin, 3-glucoside of isorhamnetin, vanillic acid, caffeic acid, chlorogenic acid, protokatechuic acid, p-coumaric acid, and syringic acid, were identified in *C. officinalis* L. extract. *C. officinalis* L. tincture containing flavonol glycosides significantly stimulated both proliferation and migration of fibroblasts in a PI3K-dependent pathway. The increase in phosphorylation of FAK (Tyr 397) and Akt (Ser 473) was detected after treatment with tincture [28].

Dinda et al. [31] obtained an interesting research result, studying the effect of *C. officinalis* L. tincture on the proliferation and migration of fibroblasts in both mouse and human models. They showed a specific signal transduction pathway in which FAK phosphorylation and subsequently activated phosphatidylinositol kinase (PI3K) regulate the healing potential of *Calendula officinalis* L. tincture. In addition, HPLC followed by ESI-MS were performed to identify key compounds present in *Calendula officinalis* L. tincture that are likely to be responsible for wound healing. Flavonol glycosides were identified as the main compounds by HPLC-MS, along with some coumarins, and amino acids. Among them, esculetin, a coumarin-class compound, was shown to stimulate type I procollagen through the PI3K/Akt and MAPK pathways in human skin. Similarly, quercetin 3-O-glucoside flavonol was also shown to have wound-healing activity. This bifunctional presence of other compounds in the tincture may be responsible for the property of wound healing.

*C. officinalis* L. also contains essential oils [26,32]. Despite a lot of studies, it is not clear which components are the most protective or stimulative for the cells’ biology. Triterpenoids were intensively investigated. Faradiol myristate and palmitate (triterpenoids) displayed comparable stimulation rates at an almost 50 μg/mL concentration, indicating that they contribute partially, but not significantly to the wound healing effects of *Calendula officinalis* L. preparations [9]. Nicolaus concluded that triterpenoids seemed to play only a marginal role, but carotene and xanthophyll derivatives should garner more attention in future studies [22].

Sesquiterpenes were the major chemical class in the examined *Calendula officinalis* L. extract. The most abundant was α-cadinene (18.1%), followed by δ-cadinene (14.9%) and γ-cadinene (8.9%). This is in agreement with a recent paper, where extracts were prepared by hydrodistillation using a Clevenger-type apparatus, and analysis was performed by GC-MS [21]. Additionally, in the present study, the basic volatile components were terpenoid alcohols: α-cadinol (16.6%) and epi-α-muurolol (11.8%). The results agreed with data obtained from the hydrodistillation technique, but were contrary to the study by Gazim et al. [33]. As in our case, the proper choice of SPME fiber allowed the identification of sesquiterpenoid alcohols. Polydimethylsiloxane/Divinylbenzene (PDMS/DVB) coating is especially recommended.

Gas chromatography and mass spectrometry (GC-MS) of 50% ethanol extract of *Calendula officinalis* L. showed that carvacrol, thymol, ethyl hexadecanoate, and viridiflorene are the main components present in the extract [26].

The content of active ingredients in *Calendula officinalis* L. extract is also connected to extraction methods. A total of 19 components were identified in the essential oil obtained by hydrodistillation GC-MS, and only 4 of them had a quantity less than 50%. In the oil extracted with organic solvent extraction, 15 compounds were defined by GC-MS and all of them had a quantity higher than 60%. Interestingly, only four out of all compounds are common to both extracts. The oil obtained with the enfleurage method contained 11 compounds defined by GC-MS with a minimal quantity of 72%. Five of them were detected in the second method and only two (α-cadinene and δ-cadinene) were detected in all samples [34].

The achieved results do not clarify the mechanism of *Calendula officinalis* L. activity, but contribute to future studies. Cadinene is described as hepatoxic, but also as an antiproliferative for cancer cells, an analgesic, and an anti-inflammatory compound.

## 5. Conclusions

*Calendula officinalis* L. extracts with different alcohol concentrations (7% and 20%) decrease alcohol cytotoxic influence on gingival fibroblasts. As a part of of further research, we plan to perform a full analysis of the obtained decoctions/macerate from *Calendula officinalis* L. (determination of volatile and non-volatile compounds) using chromatographic methods, such as LC-MS. In addition, we intend to evaluate the antioxidant capacity of the compounds contained in the aforementioned decoctions/macerate using the so-called “DPPH” radical method.

## Figures and Tables

**Table 1:** Volatile profile of *Calendula officinalis* L. flowers.

Compound	Type	Odor/Sensory Description	Area, % Decoction	Area, % Maceration Ethanol	Area, % SPME Maceration Ethanol
limonene	terpene	citrus, fruity	-	1.2	0.2
α-thujene	terpene	woody, green	-	5.0	-
α-pinene	-	-	-	3.0	-
cis-verbenol	-	-	-	0.2	-
β-ocimene	-	-	18.0	0.4	-
1.8-cineole	-	-	-	7.6	-
α-copaene	tricyclic sesquiterpene	sweet, fruity	0.2	-	1.2
α-ionone	sesquiterpenoid ketone	woody violet (floral)	19.5	-	1.6
α-humulene (α-caryophyllene)	monocyclic sesquiterpene	spicy, peppery	0.2	-	0.8
geranylacetone	sesquiterpenoid ketone	green	0.1	-	1.2
γ-muurolene	sesquiterpene	herbal woody	0.2	-	2.4
β-ionone	sesquiterpenoid ketone	woody iris (floral)	3.3	-	4.5
α-muurolene	sesquiterpene	woody	2.4	0.1	7.1
γ-cadinene	sesquiterpene	herbal woody	1.0	3.0	8.9
δ-cadinene	sesquiterpene	woody	1.4	2.1	14.9
α-cadinene	sesquiterpene	woody dry	1.5	9.0	18.1
α-calacorene	sesquiterpene	woody	0.2	-	1.6
β-oplopenone	sesquiterpene	-	0.2	-	1.1
viridiflorol	sesquiterpenoid alcohol	tropical minty	1.0	0.9	1.9
epi-α-muurolol	sesquiterpenoid alcohol	woody	1.8	5.5	11.8
α-cadinol	sesquiterpenoid alcohol	herbal woody	1.0	20.6	16.6

## Data Availability

The datasets generated during and/or analyzed during the current study are available from the corresponding author upon reasonable request.

## Supplementary Materials

The following supporting information can be downloaded at: https://www.mdpi.com/article/10.3390/life13101949/s1, Video S1: Gingival fibroblasts—7% alcohol concentration calendula extract addition; Video S2: Gingival fibroblasts—20% alcohol concentration calendula extract addition; Video S3: Gingival fibroblasts—100% alcohol concentration calendula extract addition; Video S4: Positive control—gingival fibroblasts without calendula extract addition; Video S5: Negative control.
